# Orbital Rhabdomyosarcoma With Bone Marrow Involvement: A Case Report

**DOI:** 10.7759/cureus.96086

**Published:** 2025-11-04

**Authors:** Houda El Maoudda, Othmane Zouiten, Leila Afani, Mohamed El Fadli, Rhizlane Belbaraka

**Affiliations:** 1 Department of Medical Oncology, Hôpital Universitaire de Marrakech, Marrakesh, MAR

**Keywords:** bone marrow metastasis, case report, chemotherapy, orbit malignancy, rhabdomyosarcoma (rms)

## Abstract

Rhabdomyosarcoma (RMS) is the most common primary malignant orbital tumor in children and is very rare in adults. Bone marrow (BM) infiltration is an unusual site of metastasis and is associated with a poor prognosis; therefore, management must be initiated as promptly as possible.

We report the case of a 32-year-old adult with no significant medical history, presenting with embryonal orbital RMS accompanied by BM infiltration. Clinical examination revealed decreased visual acuity with ptosis, palpebral edema, and exophthalmos, all evolving in the context of general health deterioration. Treatment consisted of chemotherapy; however, the outcome after the first cycle was unexpected. This case highlights the aggressiveness of embryonal RMS (ERMS) in adults and underscores the importance of rapid and appropriate management through a multimodal therapeutic approach.

## Introduction

Rhabdomyosarcoma (RMS) is a rare tumor; however, it represents the most common malignant soft tissue tumor in children. Approximately 10% of cases arise in the orbital region [1]. The two most frequent histological subtypes are embryonal RMS (ERMS) and alveolar RMS (ARMS) [2]. 

Typical sites of presentation include the head and neck structures, particularly the orbit, followed by the genitourinary (GU) tract and the extremities [2]. The most common clinical features are unilateral proptosis (30%), eyelid edema (21%), and blepharoptosis [3,4].

Metastatic RMS is associated with the poorest prognosis, with a three-year overall survival (OS) rate of 34% [5]. Bone marrow (BM) metastases occur in approximately 6% of RMS cases [6]. Patients with BM involvement have a three-year event-free survival (EFS) of 14%, compared with 34% in those without BM metastases (p < 0.0001) [7].

## Case presentation

Clinical findings

We report the case of a 32-year-old patient with no significant past medical history. The onset of symptoms dates back to July 2024 (five months), when the patient experienced decreased visual acuity associated with ptosis, which was initially neglected. This was followed by the development of eyelid edema and proptosis, all evolving in the context of general deterioration of health.

**Figure 1:**
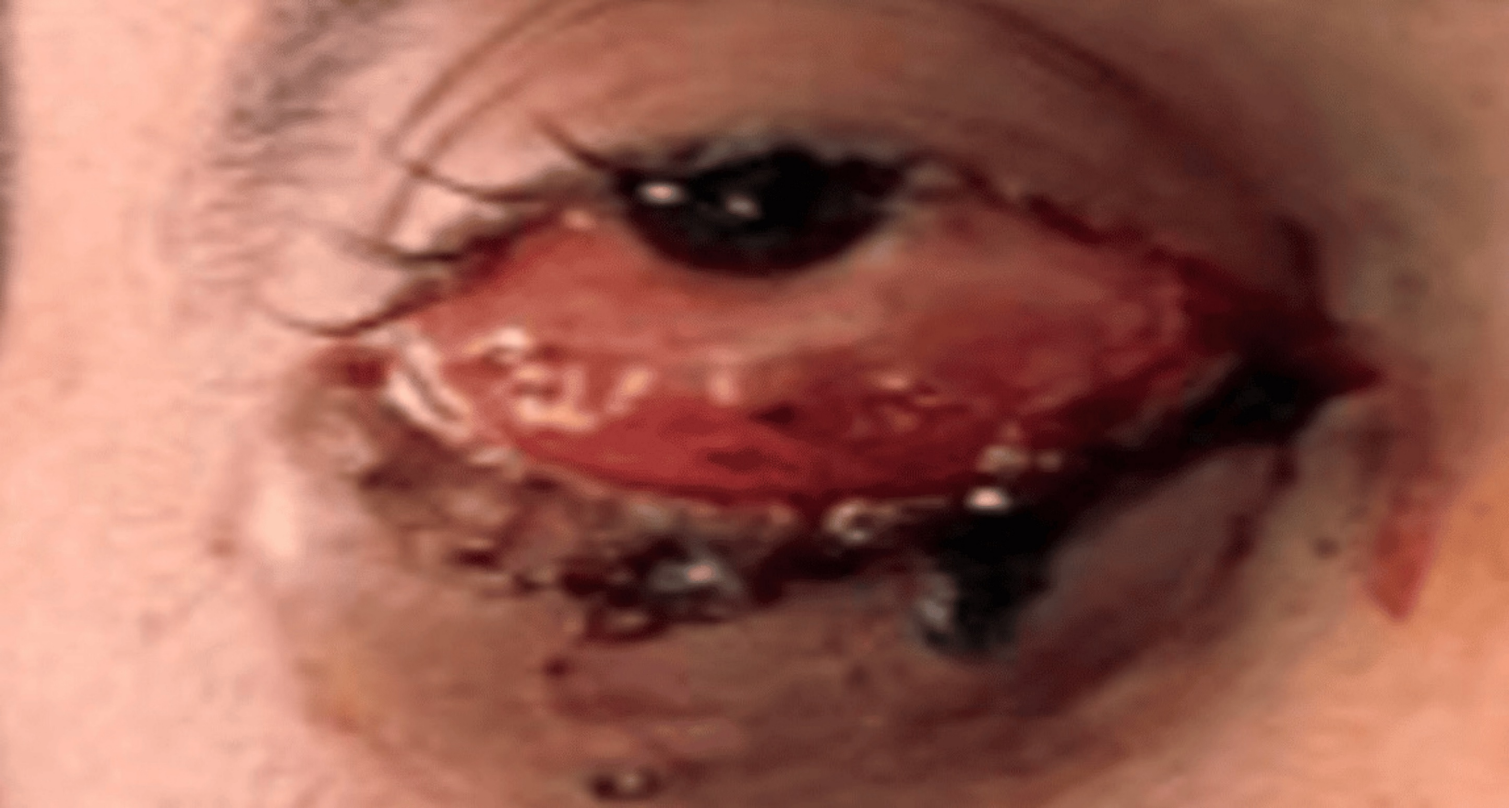
A 32-year-old adult patient presenting with right eyelid edema, non-axial exophthalmos, and ptosis

At the initial consultation, ophthalmologic examination of the right eye revealed a visual acuity of 0/10, intraocular pressure of 16 mmHg, and ptosis covering the visual axis, associated with lower eyelid swelling. The patient also presented with non-axial, inflammatory, and irreducible proptosis, inflammatory chemosis, and the presence of an intermaculopapillary fold (Figure 1).

Radiological presentation

An orbito-cerebral CT scan demonstrated a tumoral lesion centered on the floor of the right orbit, originating from the maxilla, with a suspicious radiological appearance.

**Figure 2:**
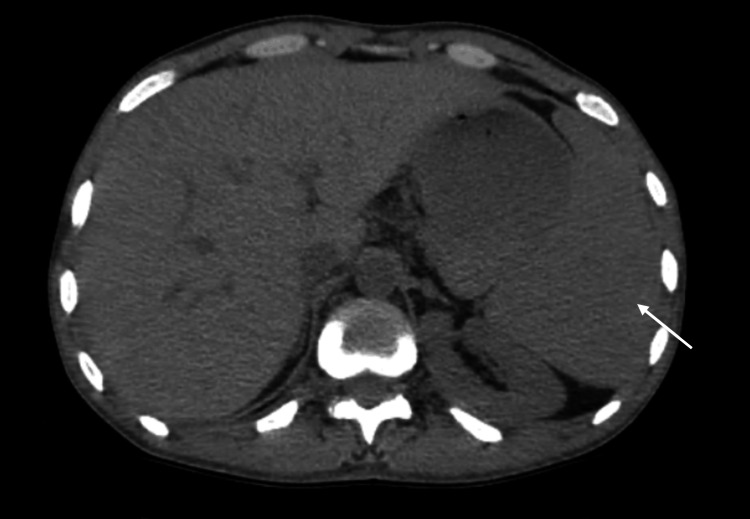
Axial cross-sectional image without contrast injection demonstrating splenomegaly (marked by the arrow)

**Figure 3:**
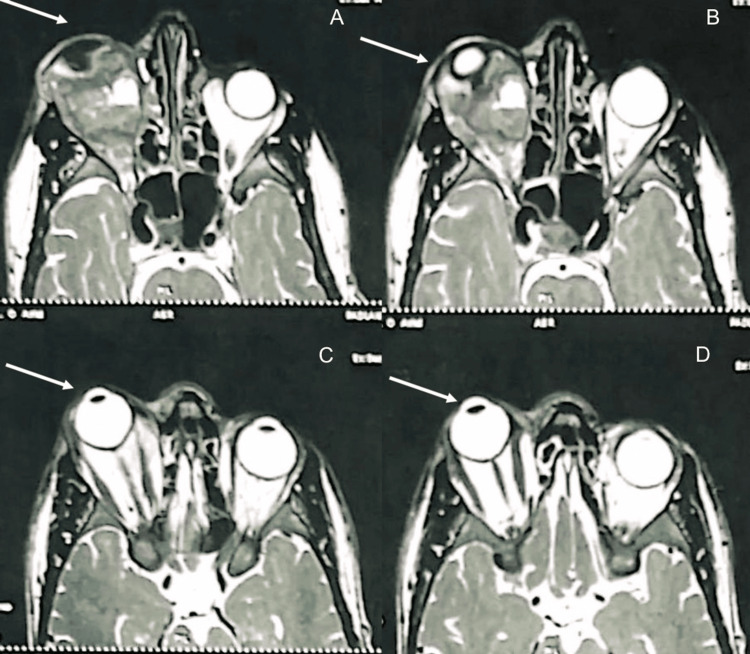
Four transverse orbital MRI images a-d: illustrating the orbital tumoral process (marked by the arrow)

Further evaluation with orbito-cerebral and facial magnetic resonance imaging revealed a right-sided soft tissue mass, located in an extra-conal intraorbital position, lytic in nature, with extension into the right maxillary sinus, right ethmoidal cells, and the right nasolacrimal canal. The lesion measured 46 × 45 × 57 mm and exhibited a heterogeneous T2 hyperintensity and a T1 hypointensity. A grade 3 ipsilateral exophthalmos was also observed, with no abnormalities detected in the cerebral parenchyma. Figures 2, 3 are imaging findings. 

Histopathology and immunohistochemistry

**Figure 4:**
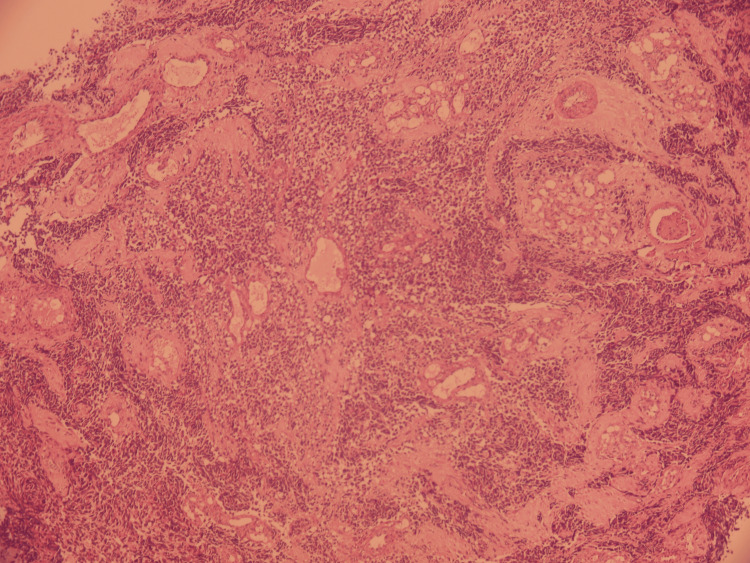
Sheets and trabeculae of round tumor cells Low magnification (×40)

**Figure 5:**
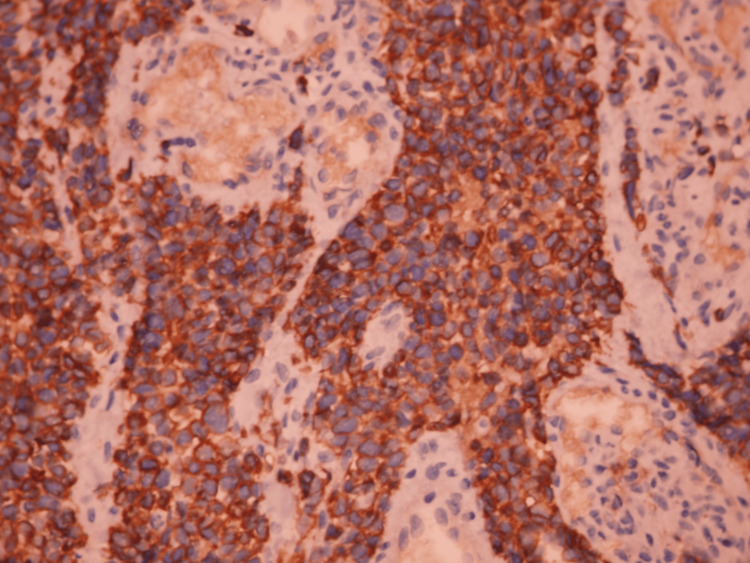
Nuclear positivity for myogenin antibody and cytoplasmic positivity for desmin antibody High magnification (×400)

Biopsy revealed a round-cell tumoral proliferation (Figure 4). Immunohistochemical analysis showed positivity for anti-myogenin and anti-desmin, while anti-pan-cytokeratin, anti-CD45 PL, and anti-CD99 were negative (Figure 5). These findings are consistent with an ERMS. Molecular testing for PAX3-FOXO1 or PAX7-FOXO1 gene fusion was not performed due to unavailability.

Results and follow-up

At admission to the medical oncology department, the patient reported left flank and left lumbar fossa pain. An orbito-cerebral CT scan revealed a homogeneous splenomegaly measuring 189 mm, with no additional abnormalities.

Laboratory investigations demonstrated severe anemia (hemoglobin 5.5 g/dL, grade 3), normochromic and normocytic, associated with thrombocytopenia (11,000/mm³, grade 4). The patient also presented moderate oral palatal bleeding, which required the transfusion of three units of packed red blood cells. An etiological workup, including a BM aspirate, confirmed the presence of medullary infiltration.

The case was discussed in a multidisciplinary tumor board meeting. The decision was to initiate first-line chemotherapy with the VAD regimen: vincristine 1.5 mg/m² and doxorubicin 60 mg/m² every two weeks, with granulocyte colony-stimulating factor (G-CSF) support.

On day 7, the patient developed febrile neutropenia complicated by sepsis of undetermined origin, which led to death despite appropriate medical management.

## Discussion

RMS is a highly malignant tumor. Orbital involvement represents 10-12% of all RMS cases and accounts for 3-4% of pediatric exophthalmos. In 71% of cases, exophthalmos is the initial clinical presentation of orbital RMS [8].

Cross-sectional imaging is essential for lesion characterization, defining topography, estimating tumor volume, and assessing locoregional extension [9,10]. On CT, these tumors usually present as well-circumscribed, homogeneous masses with moderate to marked enhancement. In our patient, CT revealed a heterogeneously enhancing mass in the right superior extraconal compartment, with loss of interface with the superior rectus muscle and a focal erosion of the superior orbital wall. Such lesions typically appear isointense to muscle on T1-weighted MRI and hyperintense on T2-weighted MRI, with moderate to marked post-contrast enhancement [11].

Histological confirmation is mandatory and is obtained through direct biopsy of the orbital mass [10-12]. Several histological subtypes have been described: embryonal type (60%), most frequent in children; alveolar type (20%), occurring in adolescents; and pleomorphic type (20%), which predominates in adults [9-13].

BM metastatic involvement occurs in about 6% of RMS cases and carries a particularly poor prognosis [6]. The three-year EFS is only 14% compared with 34% in the absence of marrow involvement (p < 0.0001) [7]. The typical profile includes an adolescent patient (median age ~16 years), an extremity tumor, and the presence of a PAX3-FOXO1 translocation [2]. Diagnosis is often delayed, with ≥3 metastatic sites at presentation [2], and is usually established through fluorodeoxyglucose (FDG)-PET [14,15]. The peculiarity of our case lies in the orbital primary tumor associated with BM infiltration, an extremely rare presentation.

Management of RMS requires rapid, multimodal therapeutic intervention, combining chemotherapy, surgery, and radiotherapy. In adults, where no consensus exists due to the absence of dedicated clinical trials, treatment is extrapolated from pediatric protocols [16]. Commonly used regimens include AEWS1031 or mesna, adriamycin, ifosfamide, and dacarbazine (MAID), but vincristine-doxorubicin-cyclophosphamide (VDC) remains a cornerstone of treatment. The advent of newer chemotherapy protocols has considerably improved outcomes, with five-year survival rates now exceeding 85% [2].

Treatment must be initiated promptly, as prognosis is closely related to diagnostic and therapeutic delays. In adults, therapeutic decisions remain guided by pediatric recommendations and retrospective adult case series [16]. Newer chemotherapy regimens have further improved prognosis, with five-year survival rates ranging from 86% to 95% [17]. Initial treatment regimens have included AEWS1031, MAID, COP, and AAML1031, after which patients were managed according to Children’s Oncology Group (COG) trials (typically ARST05319) or institutional pilot studies at Memorial Sloan Kettering (MSK) (IRB 03-09910). Treatments used have included, sequentially: vinorelbine/oral cyclophosphamide/bevacizumab, vincristine/dactinomycin, topotecan/cyclophosphamide, vincristine/topotecan/doxorubicin, or autologous tumor cell vaccine supported by IL-7 and pazopanib [2].

BM metastatic RMS carries an especially grim prognosis, with OS rates of 81%, 32%, and 20% at one, two, and three years, respectively, and a median survival of 1.5 years, lower than estimates for metastatic RMS in general [2]. The typical course includes an initial rapid remission followed by relapse in nearly 100% of cases within ~14 months [2]. While most deaths are attributed to disease progression, with only rare cases related to treatment toxicity [2], our case is distinctive in that the fatal outcome was directly attributable to post-therapeutic complications.

In the absence of treatment, survival is limited to a few months. Chemotherapy generally provides disease control for 12-16 months; however, early, multi-metastatic relapses remain uniformly fatal in the short term [2]. This consistently unfavorable course underscores the urgent need for novel strategies, including prospective clinical trials and prolonged maintenance therapies, to consolidate remissions and potentially alter the natural history of this disease.

## Conclusions

Although most RMS occur in children, they can rarely be encountered in adults, and orbital localization is even more uncommon. Prognosis is strongly influenced by the presence of metastases, particularly BM involvement, which confers an especially poor outcome. Management in such cases relies mainly on palliative chemotherapy, with limited clinical benefit. In our patient, the clinical course was unfavorable.
